# Climate change to severely impact West African basin scale irrigation in 2 °C and 1.5 °C global warming scenarios

**DOI:** 10.1038/s41598-018-32736-0

**Published:** 2018-09-26

**Authors:** Mouhamadou Bamba Sylla, Jeremy S. Pal, Aissatou Faye, Kangbeni Dimobe, Harald Kunstmann

**Affiliations:** 1 West African Science Service Center on Climate Change and Adapted Landuse (WASCAL), Competence Center, Ouagadougou, Burkina Faso; 2 Department of Civil Engineering and Environmental Science, Loyola Marymount University, Los Angeles, California, USA; 3 West African Science Service Center on Climate Change and Adapted Landuse (WASCAL), Graduate Research Program on West African Climate System, Federal University of Technology – Akure (FUTA), Akure, Nigeria; 4 International Centre for Theoretical Physics (ICTP), Earth System Physics Section, Trieste, Italy; 5 Karlsruhe Institute of Technology, Campus Alpin, Institute of Meteorology and Climate Research, Department of Atmospheric Environmental Research (IMK-IFU), Garmisch-Partenkirchen, Germany; 6 University of Augsburg, Institute of Geography, Augsburg, Germany

## Abstract

West Africa is in general limited to rainfed agriculture. It lacks irrigation opportunities and technologies that are applied in many economically developed nations. A warming climate along with an increasing population and wealth has the potential to further strain the region’s potential to meet future food needs. In this study, we investigate West Africa’s hydrological potential to increase agricultural productivity through the implementation of large-scale water storage and irrigation. A 23-member ensemble of Regional Climate Models is applied to assess changes in hydrologically relevant variables under 2 °C and 1.5 °C global warming scenarios according to the UNFCCC 2015 Conference of Parties (COP 21) agreement. Changes in crop water demand, irrigation water need, water availability and the difference between water availability and irrigation water needs, here referred as basin potential, are presented for ten major river basins covering entire West Africa. Under the 2 °C scenario, crop water demand and irrigation water needs are projected to substantially increase with the largest changes in the Sahel and Gulf of Guinea respectively. At the same time, irrigation potential, which is directly controlled by the climate, is projected to decrease even in regions where water availability increases. This indicates that West African river basins will likely face severe freshwater shortages thus limiting sustainable agriculture. We conclude a general decline in the basin-scale irrigation potential in the event of large-scale irrigation development under 2 °C global warming. Reducing the warming to 1.5 °C decreases these impacts by as much as 50%, suggesting that the region of West Africa clearly benefits from efforts of enhanced mitigation.

## Introduction

In West Africa, rainfed agriculture is the most prominent instrument for securing income and overcoming poverty^1^. Agricultural water requirements in the region are largely met by rainfall that is associated with the West African monsoon occurring in the boreal summer. Irrigated agriculture represents about 4% of the cultivated land in Sub-Saharan Africa and remains largely undeveloped due to a lack of sufficient economic resources and political will^2^. Consequentially, changes in monsoon intensity, timing and spatial patterns as well as temperatures have the potential to both positively and negatively impact agricultural productivity in West Africa and consequently the wellbeing of its population.

In recent decades, drought, population increase and water withdrawals have increased water stress in the major river basins of West Africa^3^. Previous studies of climate change impacts on crops in the region generally project yields to decrease even by 2050 due to increased growing season temperatures and changes in the monsoon precipitation variability, thereby amplifying food insecurity in an already vulnerable region^4,5^. These studies, however, are largely based on output from coarse resolution (~100 to 200 km) Couple Atmosphere-Ocean Global Climate Models (GCMs) that generally do not adequately resolve the mesoscale processes required to accurately simulate precipitation in the region, particularly in subregions with complex orography or coastlines^6^.

Regional climate models (RCMs) have had success in simulating and projecting climate change in West Africa^7–14^. However, these studies tend to be uncoordinated in the sense that they consider different emissions pathways and different future and historical time periods. In addition, they tend to focus on changes of temperature and precipitation, and not to further variables that are additionally relevant to water resources. Lastly, hypotheses of future GHG emissions pathways engender an additional level of uncertainty in the projections which is also problematic of most of the GCM studies^15,16^.

The relatively few climate change impacts studies on water resources in West Africa suffer from the same issues as the pure RCM studies, in addition to two others^17–20^. First, there is a lack of observations in many river basins, and considerable discrepancies exist in available data used to calibrate respective hydrological models^21,22^. Second, there are significant uncertainties in projected changes by climate and/or hydrological models^17,23,24^. These combined sources of uncertainties and the high vulnerability of the region due to its economic conditions make it vital to provide reliable future climate information for water resources, including irrigation potential over the major river basins using a unified approach under consistent climate change scenarios.

In this study, we aim to reduce the uncertainties resulting from the aforementioned issues by taking advantage of a 23-member high resolution ensemble of RCM simulations performed as part of the most recent high resolution COordinated Regional climate Downscaling EXperiment (CORDEX). Future warming scenarios considered here are based on the 2015 United Nations Framework Convention on Climate Change (UNFCCC) 21^st^ Conference of Parties (COP 21) Paris Agreement, where all nations pledged to hold global temperature increase to below 2 °C (relative to pre-industrial levels) and to explore further efforts to limit the increase to 1.5 °C. How the hydroclimatology of the West African major river basins will respond under such a change has yet to be investigated. This study thus evaluates and intercompares climate change impacts on the hydroclimatology of the major West African river basins under 1.5 °C and 2 °C global warming scenarios. To overcome data scarcity related to the calibration and verification of hydrological models, output from the 23-member CORDEX RCM ensemble is used to characterize key variables relevant for the hydrology and water resources over the major West African river basins^25^. Specifically, changes in water availability and crop water demand and irrigation water need are examined and intercompared to assess the irrigation potential at basin level.

## Methodology

### Regional Climate Models Experiments

**Figure 1:**
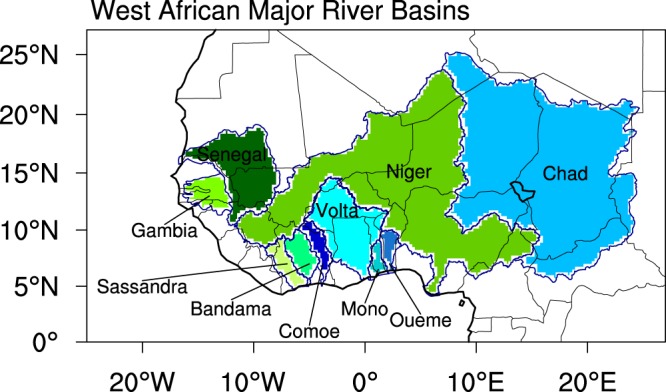
The West African domain highlighting the different major river basins.

While the study domain is West Africa, the analysis is focusing on the 10 major river basins, most of them of transboundary nature: Senegal, Gambia, Niger, Volta, Chad, Sassandra, Bandama, Comoe, Mono and Oueme (Fig. 1). The RCM simulations, performed at a 50-km grid spacing and taken from CORDEX, consist of dynamically downscaled GCM output from the Coupled Model Inter-comparison Project, Phase 5 (CMIP5)^26^ for 150-year periods based on the Representative Concentration Pathways 4.5 (RCP4.5). 10 RCMs are used, forced by various combinations of seven GCMs for a total of 22 RCM-GCM combinations (Table 1). Over West Africa, the CORDEX simulations, using both single RCMs and multi-model RCMs, have been previously evaluated^27–31^. Key findings relevant to this study include significant enhancements of the CMIP5 simulated impact-relevant precipitation and potential evapotranspiration characteristics, both in terms of magnitude and fine-scale spatial distribution^29,30,32,33^.

**Table 1:** The list of CMIP5 GCMs (see Taylor *et al*.^26^), the CORDEX RCMs (see Jones *et al*. 2011) used in this study.

	ALADIN	CanRCM	CRCM	RCA	CCLM	HIRHAM	RACMO	REMO	WRF
CCCma-CanESM2		&	&	&					
CNRM-CM5	&			&	&				
EC-EARTH				&	&	&	&	&	
GFDL-ESM2M				&					
HadGEM2-ES				&	&		&		
MPI-ESM-LR			&	&	&			&	
NCC-NorESM1-M				&		&			&

The sign “&” means that the particular RCM was used for downscaling the corresponding CMIP5 GCM.

### Derivation of Global Warming Scenarios

The 1.5 °C and 2 °C global warming scenarios are derived by extracting from each RCM simulation the 30-year period when the driving GCM projects an increase of 1.5 °C and 2 °C of global warming, respectively, compared to the pre-industrial level. Similarly, the historical reference period is taken from each RCM simulation as the 30-year period when the driving GCM simulates increase of 0.48 °C of global warming compared to the pre-industrial level. The different periods defined for 0.48 °C, 1.5 °C and 2 °C for each driving GCM are summarized in Table SI1. After the extraction of these periods from each RCM simulation, the multimodel ensemble is performed for 0.48 °C (i.e. reference period), 1.5 °C and 2 °C (future periods), respectively. Analyzing the simulations in this manner eliminates uncertainties related to emissions pathways, the choice of the baseline (i.e. the reference period) and of the future periods as well as to the propagation of the driving GCMs present-day temperature biases into the future. In addition, the large multimodel ensemble reduces uncertainty from intermodel variability and best represents the response of the climate system to an imposed forcing^34^. Therefore, the approach applied here provides a robust assessment of climate change allowing for better quantitative decision-relevant information^35^.

### Analysis Approach

The overall goal of this study is to the assess large-scale irrigation potential at a basin level in West Africa for the historical period and for the expected future climate when the global warming is limited to 2 °C and 1.5 °C. Since irrigation at the large-scale requires the development of water storage reservoirs, crop cycles would no longer need to be fully tied to the rainy season. As a result, annual mean changes are considered assuming that water can be stored and used during the dry seasons when temperatures may be more favorable depending on the location or for a second cropping cycle.

To assess the overall irrigation potential for each major river basin in West Africa, we assess crop water demand, irrigation water need, water availability, and basin irrigation potential, as follows:

#### Crop Water Demand (CWD)

CWD is considered as the evapotranspiration rate *ET*_*o*_ under well-watered conditions. It can be considered as a proxy for the water required for an optimal growth for a reference crop that completely covers the soil, is kept short, well-watered and is growing under optimal agronomic conditions^36^. While each individual crop type can have higher or lower water requirements, this is generally accepted as a reference value. In practice, this reference value is multiplied by a crop coefficient that increases or decreases the crop water requirements based on the specific crop type and growth stage. However, in West Africa, most grown cereal crops have a total crop coefficient between ~0.8 and ~1, very close to that of the reference crop Alfalfa during their growing season. Therefore CWD for the reference crop can provides a general understanding on how crop water demand is projected to change in future climate over West Africa.

Here, CWD is estimated by applying two commonly used *ET*_*o*_ formulations: Hamon^37^ and Hargreaves^36^. The Hamon formulation is based on day length, surface air temperature and saturated vapor pressure, whereas the Hargreaves formulation is a function of minimum and maximum temperatures (see SI for more details).

#### Irrigation Water Need (IWN)

IWN is water amount required in addition to effective rainfall mandatory to optimally sustain a crop. It is considered as the deficit, if any, between CWD and the effective rainfall for optimal crop growth assuming good soil conditions. Effective rainfall is considered as the portion of total rainfall available for plant growth. In this study, it is assumed that the actual evapotranspiration rate *ET* is the water used in the crop growth process and hence equivalent to the effective rainfall^36^. Therefore, IWN is simplified as the difference between *ET*_0_ and *ET*. If effective rainfall (*ET*) exceeds CWD (*ET*_*o*_), IWN is 0 and in the case of no effective rainfall, IWN is *ET*_0_ (see SI for more details).

#### Water Availability (WA)

Runoff is considered as a proxy of the water available for crop growth and is computed as difference between actual precipitation and actual *ET*. Changes in soil water storage are negligible for the time scales considered in this study.

#### Basin Irrigation Potential (BIP)

BIP provides a measure of water availability in excess or deficit of IWN that has the potential to be stored and used for irrigation. It is calculated as the difference between WA and IWN. If WA is greater than IWN, it is assumed that the basin can potentially meet its irrigation needs through the development of its water resources and vice versa. Therefore, a larger BIP in a future climate is indicative of a basin that has the potential to store more water (see SI for more details).

Since the development of water storage systems would potentially allow farmers to utilize water resources outside of the rainy season, the analysis is performed over the entire year and not just limited to the monsoon or growing seasons. This assumes a best-case scenario in the sense that West Africa in the future will improve its water resources infrastructure to best utilize its surface water and ground water resources to compensate for seasonal and inter-seasonal changes in rainfall. Annual changes are calculated as the difference between the future scenarios (1.5 °C or 2 °C global warming) minus the reference period (0.48 °C global warming). While future changes are expressed as percent of reference period values, the difference between the changes of the two scenarios are expressed as percent of the 1.5 °C scenario value.

Each of the aforementioned variables is first computed for each of the CORDEX RCM ensemble member at each grid point in a basin and then multimodel ensemble and averaging is performed. The Student’s t-test is used to characterize significant changes at a 90% confidence level.

More details about the methodology are included in the Supplementary Information (SI).

## Results

### Crop Water Demand and Irrigation Need

**Figure 2:**
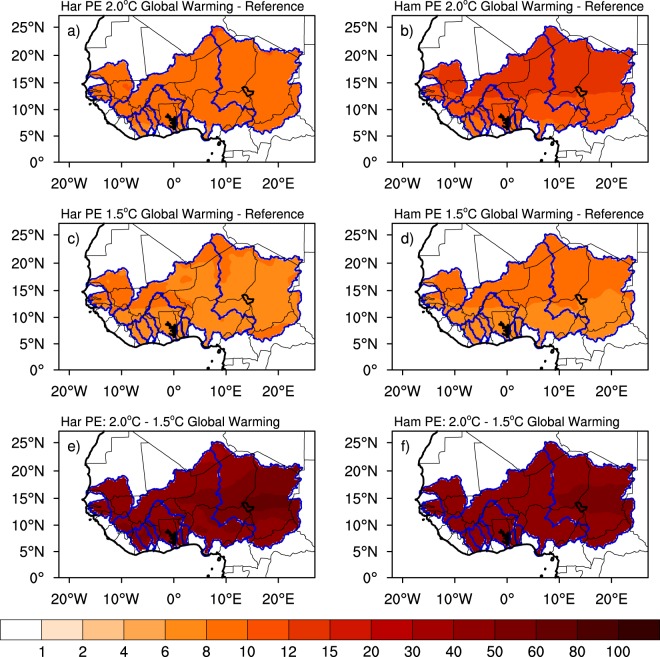
Projected changes in Potential Evapotranspiration (PET) using the methods of Hargreaves (Har; left panels) and Hamon (Ham; right panels) for 2 °C global warming scenario (upper panels), 1.5 °C global warming scenario (middle panels) and the difference between the two scenarios (lower panels). Changes for 2 °C and 1.5 °C are expressed as a percent of reference period values. Differences between the changes of the two scenarios are expressed as percent of the 1.5 °C ones. Only areas where changes are significant at 90% are shaded.

CWD is related to the amount of water needed by a crop for optimal growth and is defined here as *ET*_0_. While in reality, some crops experience CWD higher than ET_0_ and others less, this analysis provides a general understanding of how demands are projected to change in a warmer climate. Two methods (Hamon and Hargreaves) are used here to assess the projected change for the sake of robustness (see Analysis Approach). In the 2 °C global warming scenario, warmer temperatures result in an average increase in CWD by up to 10% to 15% with respect to the reference period in all major West African river basins (Fig. 2). While the Hargreaves formulation shows uniform changes throughout all basins (8% to 10%), the Hamon formulation shows more spatial variability. For example, the semi-arid Senegal, Niger and Chad basins of Sahel (north of 10°N) experience changes up to 15% and the more humid Sassandra, Bandama, Comoe, Mono, Oueme, southern Volta and Lower Niger basins of the Gulf of Guinea (south of 10°N) experience changes lower than 12%. If the future global warming is held to 1.5 °C, the projected changes are reduced by as much as 50% with the smallest changes in the Gulf of Guinea basins. It is worth noting that, although CWD increases in both *ET*_*o*_ formulations, that of Hamon generally simulates higher changes than that of Hargreaves due its higher known sensitivity to temperature changes^38,39^).

As most crops are rainfed in West Africa, the increased CWD combined with relatively small changes in precipitation (see Fig. SI1) would increase the potential for plant stress and reduce yields^40^. In fact, a recent study based on the *Inter-Sectoral Impact Model Intercomparison Project* (ISIMIP) fast-track global gridded crop models shows a reduction over West Africa of some cereals yields under both 1.5 °C and 2 °C global warming scenarios^41^. Similarly, more recent studies^42,43^ project substantial yield losses in the region irrespective of intensification case and for both scenarios of global warming.

**Figure 3:**
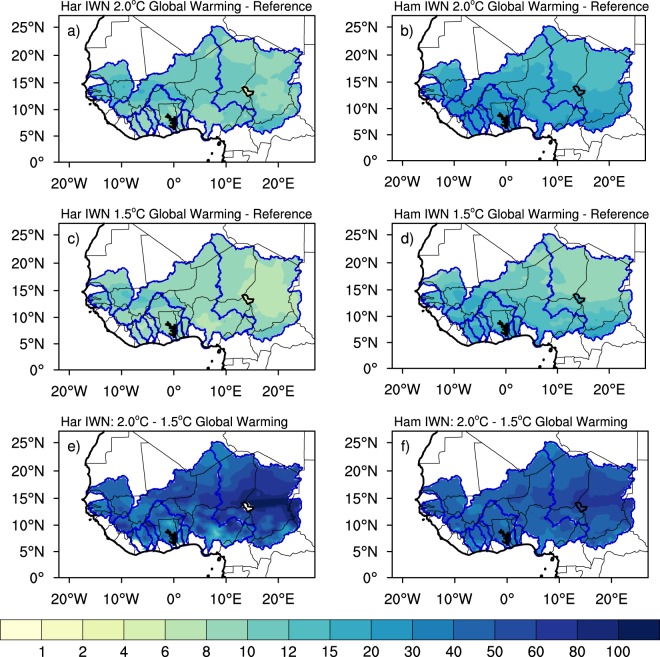
Projected changes in Irrigation Water Need (IWN) using PET from the methods of Hargreaves (Har; left panels) and Hamon (Ham; right panels) for 2 °C global warming scenario (upper panels), 1.5 °C global warming scenario(middle panels) and the difference between the two scenarios (lower panels). Changes for 2 °C and 1.5 °C are expressed as a percent of reference period values. Differences between the changes of the two scenarios are expressed as percent of the 1.5 °C ones. Only areas where changes are significant at 90% are shaded.

As indicated above, CWD in West Africa is generally met through precipitation (i.e. effective precipitation). Deficits in CWD could potentially be mitigated through the development of large-scale irrigation. IWN measures crop water needs under optimal growing conditions solely in terms of additional water requirements and provided that other conditions are optimal (e.g. diseases and pest free, favorable soil conditions). Under the 2 °C global warming scenario, IWN is projected to increase by more than 15% and 30% of reference period values, respectively, for the Hargreaves and Hamon formulations over most basins (Fig. 3). While the Hargreaves formulation displays the greatest changes (up to 20%) in western basins (i.e. Gambia, southern Senegal and Western Niger), Hamon formulation also shows extensive changes (up to 30%) in the Gulf of Guinea basins (Sassandra, Bandama and Volta). These spatial patterns of the changes differ from those of CWD. Furthermore, the increased CWD outweighs the smaller changes in *ET*_0_ irrespective of the formulation resulting in an overall increase of IWN. Limiting global warming to 1.5 °C reduces the increase to 10–20% over these regions. Although IWN is largely projected to increase over the region, it is not obvious whether future water availability will compensate for or exasperate these increases.

### Projected Water Availability and Basin Irrigation Potential

**Figure 4:**
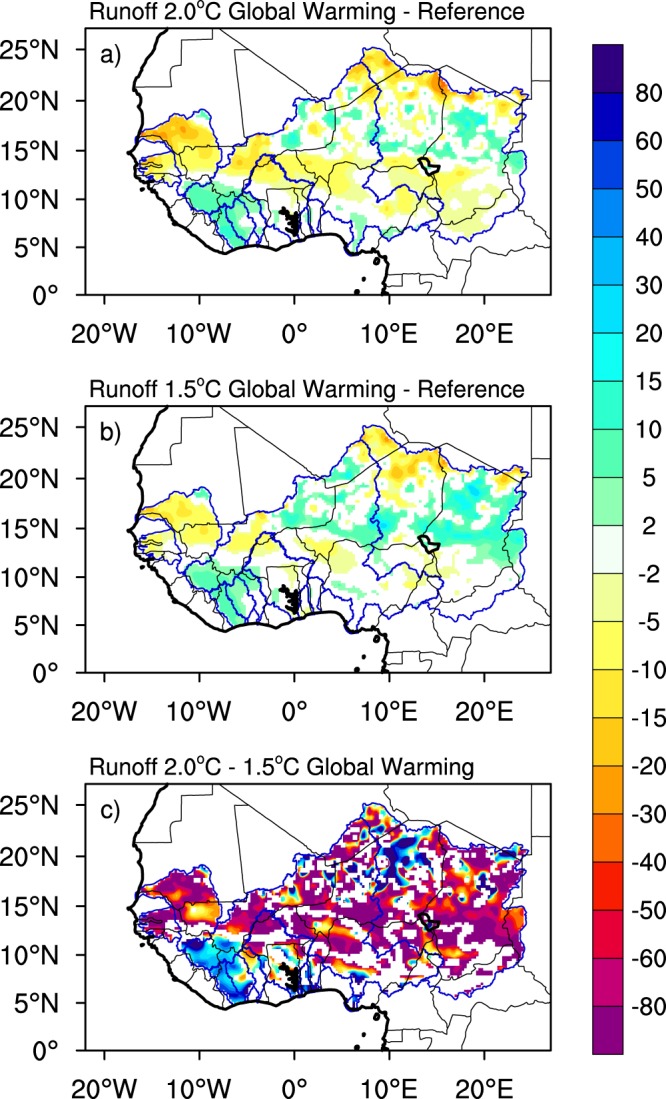
Projected changes in Total Runoff for 2 °C global warming scenario (upper panels), 1.5 °C global warming scenario (middle panels) and the difference between the two scenarios (lower panels). Changes for 2 °C and 1.5 °C are expressed as a percent of reference period values. Differences between the changes of the two scenarios are expressed as percent of the 1.5 °C ones. Only areas where changes are significant at 90% are shaded.

The projected WA changes under 2 °C of global warming are regionally heterogeneous with both significant increases and decreases depending on the region (Fig. 4). On one hand, in Senegal, Gambia, central Niger, northern Volta and northern and southern Chad, decreased precipitation (see Fig. SI1) and *ET* (see Fig. SI2) combine to reduce WA up to 30% compared to the reference period. On the other hand, in the smaller basins of the Gulf of Guinea, such as Sassandra, Bandama, Comoe, as well as northern Niger and central Chad, the increased precipitation overcompensates the increased *ET*, resulting in WA increases by up to 30%. These patterns of changes are similar to those from previous studies^41,44^ who found total runoff increases in some portions of the Sahel (i.e. Niger) and decreases in western regions of West Africa coinciding with the Senegal and Gambia basins. This result is also consistent with Gosling *et al*.^45^ who project positive changes in the upper Niger basin. Limiting global warming to 1.5 °C, decreases the negative changes and their spatial extent and strengthens and increases the spatial extent of positive changes in the Chad and Niger basins by as much as 60%. In addition, in Senegal and Gambia, the magnitude of negative changes is limited to 15% and in Sassandra, Bandama and the adjacent section of the Niger basin, the increases are limited to 10%.

**Figure 5:**
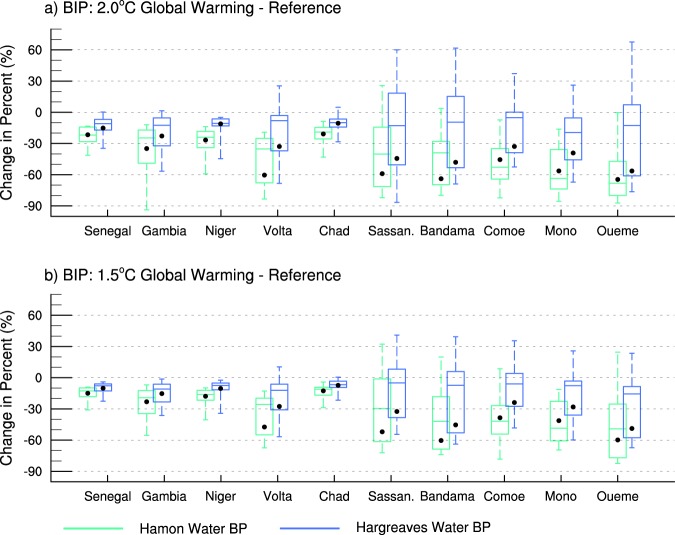
Box-Whisker-Plots of projected changes in Basin Irrigation Potential (BIP) for the 2 °C global warming scenario (upper panels) and the 1.5 °C global warming scenario (lower panels). Changes for 2 °C and 1.5 °C are expressed as a percent of reference period values. Differences between the changes of the two scenarios are expressed as percent of the 1.5 °C ones.

To examine whether the water availability is in excess or deficit compared to the IWN over each basin, BIP is considered, which provides an indication of whether or not a basin can store more water to meet demands. Despite the increases in WA in some areas, decreases in BIP is projected for all basins and both models of *ET*_0_ under the 2 °C global warming scenario (Fig. 5). In the Sahelian basins, such as Senegal, Gambia, Niger, Chad and to some extent Volta, the decreases are limited to around 10% to 30%. However, in the smaller basins located in the Gulf of Guinea (i.e. Sassandra, Bandama, Comoe, Mono and Oueme), the decrease is considerably larger ranging between 35% to 65% with the highest decreases in Bandama and Oueme. This is largely a consequence of greater changes in IWN outweighing the increases in WA in many areas of these basins. Similar results are found for the 1.5 °C scenarios but with lower values (within 15% for the Sahel basins and between 25% to 60% in the Gulf of Guinea basins). The magnitude of the changes with the Hamon formulation is greater than that of Hargreaves due to the larger IWN changes (i.e. Fig. 3). An uncertainty analysis of the ensemble members reveals that in most of the basins, the inter-quantile ranges, median and most of the distribution of the projected BIP changes are negative. This indicates that although the individual ensemble members differ compared to the ensemble average, most of the experiments project decreases in BIP suggesting that the results are robust. Furthermore, the intermodel spread is larger in the basins along the Gulf of Guinea compared to that in the Sahel hinting more consistent changes in basins such Senegal, Gambia, Niger, Chad and to some extent Volta.

Overall, the results indicate that compared to the reference period, the 10 major West African river basins will likely face considerable challenges to meet future water demands under global warming with the Gulf of Guinea basins experiencing the largest deficit. While irrigation is an important adaptation measure to climate change, these changes also imply that, future demands will likely require substantially increased water withdrawals.

## Discussion

In this study we assessed the hydroclimatology of the 10 major West African river basins under 1.5 °C and 2 °C global warming scenarios using a comprehensive ensemble of CORDEX regional climate model experiments. The results indicate that, under a relatively moderate rise of global temperature of 2 °C, the combined increases in CWD and smaller mixed changes in *ET* cause IWN to increase considerably regardless of the *ET*_0_ formulation. Such increases outweigh the projected WA changes even in regions where WA increases. For example, in western Gulf of Guinea, Niger and Central, where WA increases, changes in IWN are greater. This implies that the projected WA will not be sufficient to compensate for the projected IWN. Therefore, as climate warms, the potential to sustain irrigated agriculture or other activities that require large amounts of water such as hydropower is projected to decrease. A relatively small decrease in global warming from 2 °C to 1.5 °C significantly reduces the gap between IWN and WA greatly reducing the negative consequences of climate change on water resources. It should be emphasized that although some uncertainties are present in these projections, they are mostly in the magnitude of the changes as most of the models project negative changes.

It is worth mentioning that increased CO_2_ concentration in future climate could increase water use efficiency of plants. However, this is not taken into account in this study.

As population and urbanization intensify and economies grow, increasing municipal and industrial water needs are likely to amplify the overall water demands in West Africa. This study thus suggests that effective mitigation should be pursued to cap the global warming at 1.5 °C hoped for by COP21. Notwithstanding, West Africa will likely continue to face water limitations under climate change.

Adaptation measures should be well thought out and should consider concerted and intensified efforts (1) for the construction of surface and groundwater reservoirs to store water efficiently for irrigation, (2) for the implementation of efficient irrigation practices, and (3) for considering seasonal shifts in crop strategies to relatively cooler periods in the year.

## Electronic supplementary material

Supporting Information
