# Supplementary material for: Climate change to severely impact West African basin scale irrigation in 2 °C and 1.5 °C global warming scenarios
Source: Sci Rep. 2018 Sep 26;8:14395. doi: 10.1038/s41598-018-32736-0 (PMC6158170; doi:10.1038/s41598-018-32736-0)
Supplement: Supplementary file 1 — Supporting Information [file 41598_2018_32736_MOESM1_ESM.pdf]

Supporting Information for

Climate change to severely impact West African basin scale irrigation in 2ºC and 1.5ºC global warming scenarios

Mouhamadou Bamba Sylla*[1], Jeremy S Pal[2], Aissatou Faye[1,3,4], Dimobe Kangbeni[1], Harald Kunstmann[5,6]

1. West African Science Service Center on Climate Change and Adapted Landuse (WASCAL), Competence Center, Ouagadougou, Burkina Faso
2. Department of Civil Engineering and Environmental Science, Loyola Marymount University, Los Angeles, California, USA
3. West African Science Service Center on Climate Change and Adapted Landuse (WASCAL), Graduate Research Program on West African Climate System, Federal University of Technology – Akure (FUTA), Nigeria
4. International Centre for Theoretical Physics (ICTP), Earth System Physics Section, Trieste, Italy
5. Karlsruhe Institute of Technology, Campus Alpin, Institute of Meteorology and Climate Research, Department of Atmospheric Environmental Research (IMK-IFU), Garmisch-Partenkirchen, Germany
6. University of Augsburg, Institute of Geography, Augsburg, Germany

*Corresponding author:** Mouhamadou Bamba Sylla (syllabamba@yahoo.fr; sylla.b@wascal.org)

1. Reference, 1.5°C and 2°C of global warming periods

To identify the reference period and the two future periods, the 30-year when the driving GCM simulates increase of 0.48°C, *1.5°C and 2°C* of global warming compared to the pre-industrial level is extracted for each experiment. The different periods for each of the GCMs are summarized in Table SI1.

Table SI1: *Summary of reference (i.e. 0.48°C), 1.5°C and 2°C of global warming periods for each GCM*

GCMs	Reference Period (0.48°C)	Future Period (1.5°C)	Future Period (2°C)
CanESM2	1969-1998	2006-2035	2018-2047
HadGEM2-ES	1984-2013	2017-2046	2032-2061
MPI-ESM-LR	1958-1987	2006-2035	2031-2060
CNRM-CM5	1974-2003	2021-2050	2043-2072
NorESM1-M	1976-2005	2029-2058	2064-2093
GFDL-ESM2M	1971-2000	2032-2061	2071-2100
EC-EARTH	1958-1987	2008-2037	2030-2059

2. Derivation of the Variables

- Crop Water Demand (CWD):

CWD is calculated as Potential Evapotranspiration (*PE* or ET_0). It is defined as the amount of water that would be evaporated and transpirated if there were sufficient water available. It represents the water required by the crop for optimal growth. Here, ET_0 is computed using two commonly used formulations: Hamon (1963) and Hargreaves (FAO 1998). ET_0 in mm/day according to the Hamon formulation is based on the daylength *D* in hours, the surface air temperature *T (°C)* and saturated vapor pressure $E_S(T)$ in *kPa* at *T* as follows:

$$ET_0 = \frac{715.5 * D * E_S(T)}{T + 273.2} \qquad (1)$$

$E_S(T)$ is calculated according to the Tetens (1930) method:

$$E_S(T) = 0.611 * ln\left[\frac{17.67/T}{T + 243.5}\right] \qquad (2)$$

ET_0 in the Hargreaves formulation is a function of the mean, minimum, and maximum tempratures, T_{mean}, T_{max} and T_{min}, respectively, expressed in ^oC as well as the extraterrestrial radiation (in *mm/day*):

$$ET_0 = 0.0023 * (T_{mean} + 17.8) * (T_{max} - T_{min})^{0.5} R_a \quad (3)$$

It should be noted that this is the crop water need for a mature reference crop of *alfalfa*. The actual CWD are typically varied according to the crop type and stage of growth.

- Irrigation Water Need (IWN):

Irrigation Water Need (IWN) is considered as the deficit, if any, between CWD and the effective rainfall for optimal crop growth assuming good soil conditions. In other words, any CWD not met by effective rainfall should be compensated for with irrigation for optimal growth. Effective rainfall is considered the portion of total rainfall available for plant (i.e. actual evapotranspiration or *ET).* If effective rainfall exceeds CWD, IWN is 0 and in the case of no rainfall, IWN is ET_0. In all other cases, IWN is computed as follows:

$$IWN = ET_0 - ET \quad (4)$$

- Water availability (Runoff)

The water balance equation can be written as follow:

$$P = ET + RO + S \quad (5)$$

Where *P* is precipitation, *ET* is evapotranspiration, *RO* is runoff and *S* change in storage. For the long-term (annual or more), the change in storage is negligible. For the purpose of this study and considering 30 years of climatology, the water balance can be written as follow:

$$RO = P - ET \quad (6)$$

Therefore, the runoff is calculated as the difference between Precipitation and Evapotranspiration and is here considered as a proxy for water availability.

- Basin Irrigation Potential (BIP):

BIP measures water availability in excess or deficit of IWN. It is calculated as the difference between Runoff (or water availability) and IWN.

$$BIP = RO - IWN \quad (7)$$

If runoff is greater than IWN, the basin is likely to gain more water. When runoff is smaller than IWN the basin is likely to loose water (the amount to be withdrawn and used for irrigation). Therefore a larger BIP in future climate describes a basin that will likely stores more water compared to the reference period and sustain irrigation activities. A smaller BIP reflects a basin that will likely stores lesser water with respect to the reference period.

3. Projected Precipitation and Evapotranspiration (i.e. Effective Precipitation)

Projected precipitation and evapotranspiration show similar patterns of changes (Fig SI1-2). In fact for both scenarios of global warming, precipitation and evapotranspiration decrease in the Sahel, i.e. in Senegal Gambia, northern Niger, northeastern Chad by up to 15% and increase in the basins along the Gulf of Guinea as well as central and eastern Chad for about 20% compared to the reference period. Compared to the 1.5^{o}C case, the 0.5^{o}C additional warming strengthens the drying in the Sahelian basins. In addition, it decreases the wet conditions as well as their area extent in central and eastern Chad while substantially increases them in most of the basins in the Gulf of Guinea.

Supplemental Figures

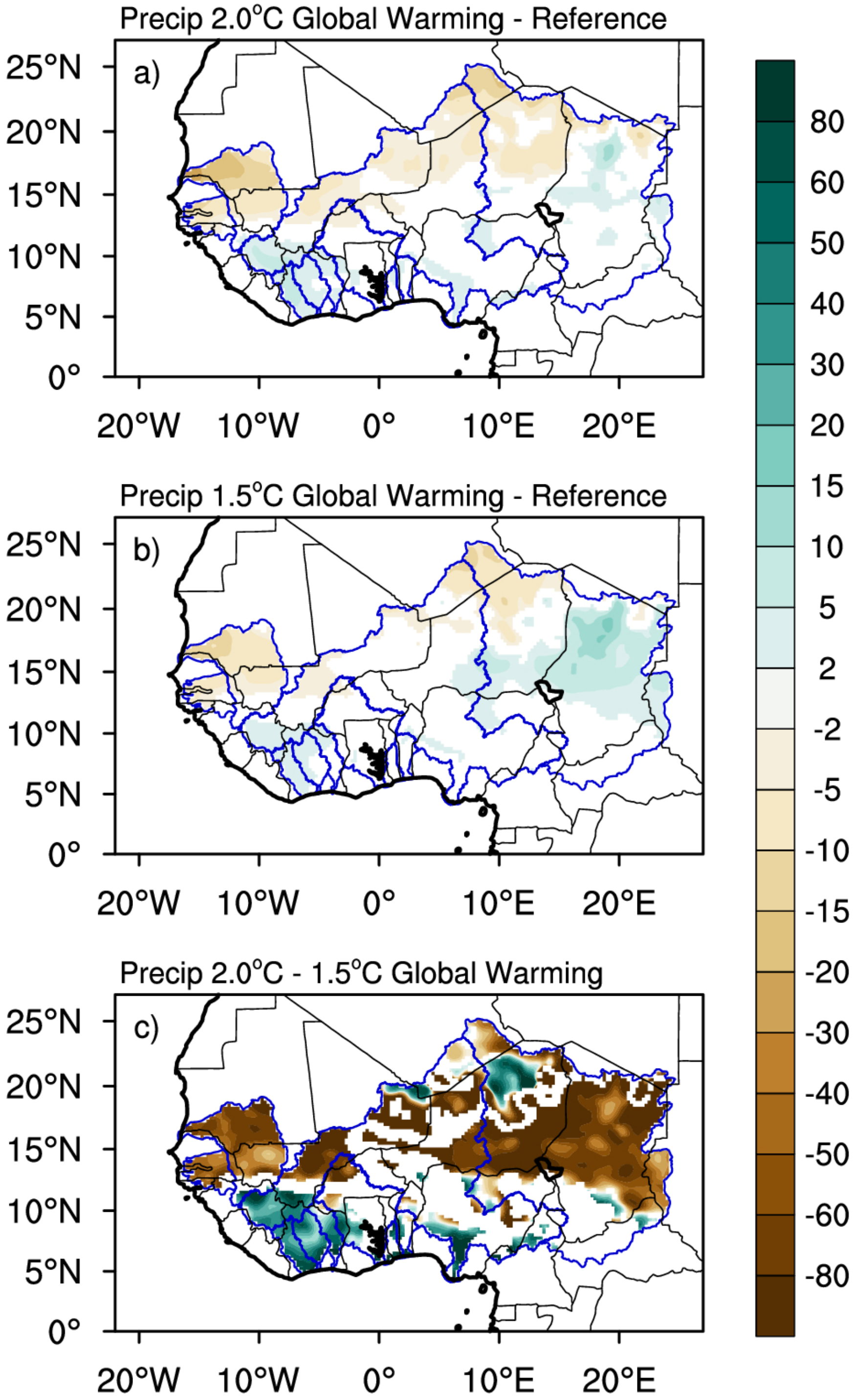

Figure SI1: *Projected changes (Future minus Reference) in precipitation for the 2°C global warming scenario (upper panel), the 1.5°C global warming scenario (middle panel) and the difference between the two scenarios (lower panel).Changes for 2°C and 1.5°C are expressed as a percent of reference period values. Differences between the changes of the two scenarios are expressed as percent of the 1.5°C ones. Only areas where changes are significant at 95% are shaded*

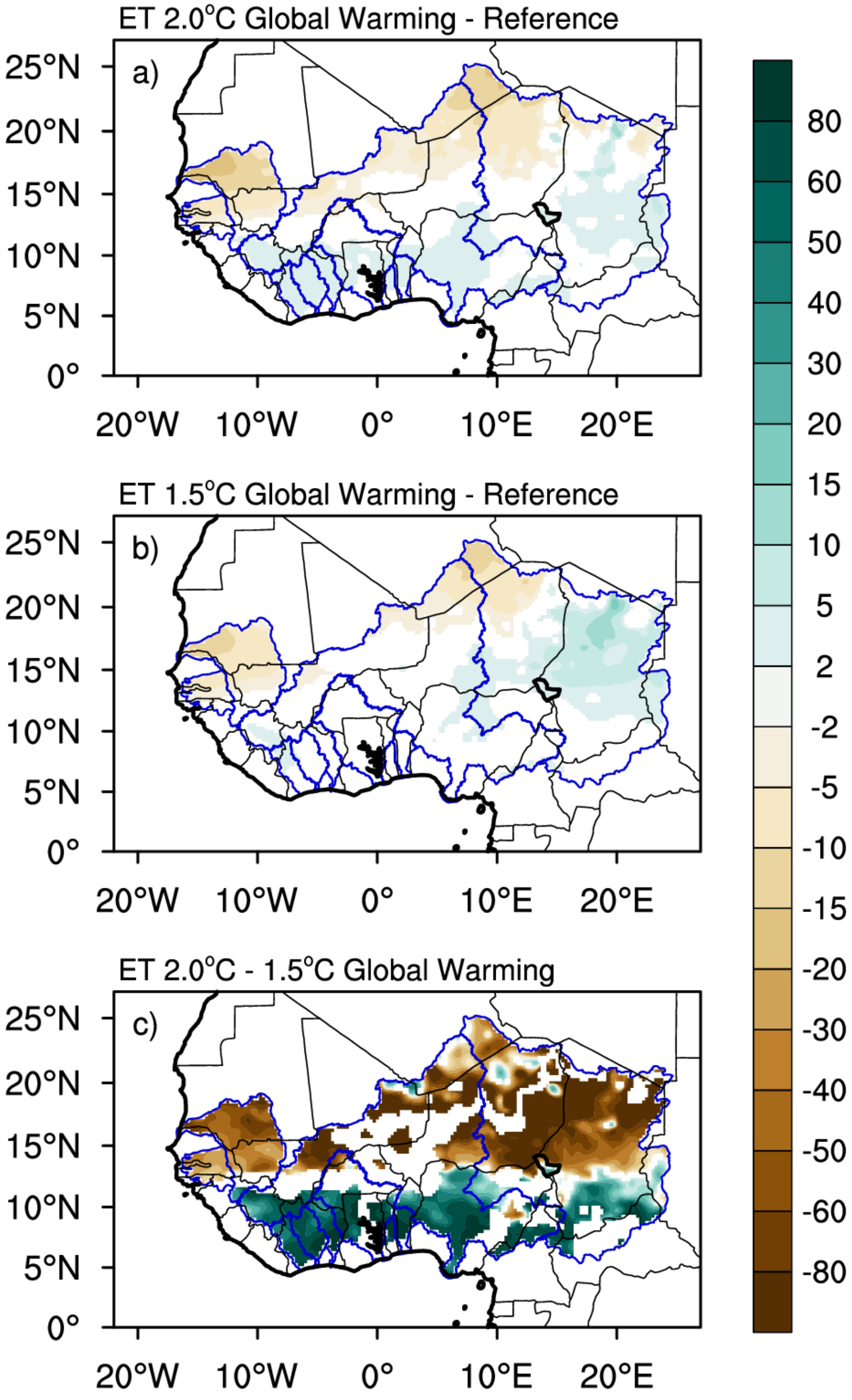

Figure SI2: *Projected changes (Future minus Reference) in evapotranspiration for the 2°C global warming scenario (upper panel), the 1.5°C global warming scenario (middle panel) and the difference between the two scenarios (lower panel).Changes for 2°C and 1.5°C are expressed as a percent of reference period values. Differences between the changes of the two scenarios are expressed as percent of the 1.5°C ones. Only areas where changes are significant at 95% are shaded*
